# Prolonged Shedding of Zika Virus RNA in Vaginal Secretions, Nicaragua 

**DOI:** 10.3201/eid2504.180977

**Published:** 2019-04

**Authors:** Yaoska Reyes, Natalie M. Bowman, Sylvia Becker-Dreps, Edwing Centeno, Matthew H. Collins, Guei-Jiun Alice Liou, Filemón Bucardo

**Affiliations:** Universidad Nacional Autónoma de Nicaragua, León, Nicaragua (Y. Reyes, E. Centeno, F. Bucardo);; University of North Carolina, Chapel Hill, North Carolina, USA (N.M. Bowman, S. Becker-Dreps, M.H. Collins, G.-J.A. Liou)

**Keywords:** Zika virus, viruses, transmission, shedding, Nicaragua, vaginal secretions, sexually transmitted diseases

## Abstract

Zika virus, an arthropod-borne flavivirus pathogen in humans, is unusual because it can be sexually transmitted and can be shed for prolonged periods in semen. We report viral shedding in vaginal secretions for up to 6 months, indicating the potential for sexual and vertical transmission by infected women.

Zika virus swept through the Americas beginning in 2014, with >1 million cases reported through December 2017 (Pan American Health Organization, https://www.paho.org/hq/index.php?option=com_docman&task=doc_view&Itemid=270&gid=43297&lang=en). Zika virus had been considered relatively benign, but during the Americas epidemic, several new pathogenic features were identified: association with severe birth defects in infants born to women infected during pregnancy, transmission by sexual contact, prolonged shedding in semen, and Guillain-Barré syndrome (*1*–*3*). Shedding in the female genitourinary tract has not been widely reported and remains a serious question because of the potential for sexual transmission or ascending fetal infection in a pregnant woman (*4*).

## The Study

During October 2016–November 2017, we recruited women >18 years of age with symptomatic Zika virus infection (rash, fever, and/any combination of conjunctivitis for <7 days) at a public health center and Hospital Escuela Oscar Danilo Rosales Argüello in León, Nicaragua. We enrolled 5 women in a prospective cohort to characterize duration of viral shedding in the genital tract. We collected blood by venipuncture, and each woman provided urine and saliva samples.

We tested whole blood, urine, and saliva from each woman for Zika virus RNA at enrollment with quantitative reverse transcription PCR (qRT-PCR). If we detected Zika virus RNA in any fluid, we asked women to provide blood, urine, saliva, and vaginal secretions at 7, 14, 21, 28, 60, 90, and 180 days postonset (dpo) of initial symptoms. We collected vaginal fluid using the BBL Culture Swab Collection and Transport System (Becton Dickinson, http://www.bd.com). Vaginal swabs were self-collected by the woman, who inserted the swab ≈5 cm into the vagina, left it in place for 5 minutes, removed it, and placed it into swab transport media. Women did not provide samples during menstruation to avoid contamination with blood. Study staff transported the swab specimens at 4°C within 3 hours to the Universidad Nacional Autónoma de Nicaragua-León microbiology laboratory, where it was stored at 4°C for up to 4 months until RNA extraction.

To elute Zika virus RNA, we incubated vaginal swabs in 140 μL of phosphate-buffered saline at room temperature for 10 min. We extracted Zika virus RNA from the eluent using the QIAamp Viral RNA Mini Kit (QIAGEN, https://www.qiagen.com) according to manufacturer instructions. We detected Zika virus RNA by qPCR (AgPath-ID, Applied Biosystems, https://www.thermofisher.com) on an ABI 7500 RT-PCR system (Applied Biosystems) using published primers that detect all known Zika virus genotypes (Zika1087/1108FAM/1163c) and those that are specific to the Asian genotype (Zika4481/4507cFAM/4552c) (*5*,*6*). We added 8 μL RNA to 12 μL 2X RT-PCR buffer, 1 μL (10 pmol) each forward and reverse primers, 1 μL (10 pmol) probe, 1 μL 25X RT-PCR enzyme mix, and 1.7 μL detection enhancer to a final volume of 25 μL. We amplified Zika virus RNA at 50°C for 30 min and 95°C for 15 min, followed by 44 cycles of 95°C for 15 s and 60°C for 1 min. We used QIAGEN AVE buffer as negative RNA purification control and DEPC water as no-template control. The positive control was cell culture supernatant of the FP/2013 strain. We defined a positive sample as a cycle threshold (C_t_) <38 in either reaction.

The study was approved by the institutional review boards of Universidad Nacional Autónoma de Nicaragua-León (acta no. 86, 2016) and University of North Carolina at Chapel Hill (protocol no. 16–0541). All subjects provided written informed consent at enrollment.

Five women with acute Zika virus infection provided >1 sample of vaginal secretions. The median age of these participants was 25 years (range 18–54 years). Four women were pregnant; gestational age at enrollment was 1–8 months. Infants of all 4 women were healthy at birth with no obvious congenital anomalies. The 54-year-old subject had hypertension; no other participants reported chronic medical conditions. Symptoms started a median of 4 days before enrollment (range 1–6 days).

No woman provided samples at all time points because of menstruation or lack of follow-up. Of 18 vaginal fluid specimens examined, 10 (56%) were Zika virus positive by qRT-PCR with >1 of the primer sets, and 3 were positive by both. Neither sample collected at 7 dpo was qRT-PCR positive, but 2/3 (67%) were Zika virus positive at 14 dpo, 3/4 (75%) at 21 dpo, 1/3 (33%) at 28 dpo, 2/2 (100%) at 60 dpo, 1/2 (50%) at 90 dpo, and 1/2 (50%) at 180 dpo. Three women had only 1 vaginal fluid sample positive for Zika virus by qRT-PCR; 1 had 2 of 3 positive; and 1 woman, who shed through 180 dpo, had Zika virus in 5 of 6 samples tested (Table).

**Table:** Clinical characteristics and viral shedding in 5 women with acute Zika virus infection, Nicaragua, 2016–2017*

Characteristic	A013	A018	A020	A024	A039
Age, y	54	18	25	19	25
Duration of symptoms at enrollment, d	1	3	4	6	5
Pregnant at enrollment	No	Yes	Yes	Yes	Yes
Estimated gestational age at enrollment, mo	NA	7	7	5	1
Timing of delivery, days after symptom onset	NA	39	61	149	305
Hematocrit, %, reference range 36–45	36	32	35	37	40
Leukocytes, cells/mm^3^, reference range 5,000–10,000	4,230	5,410	3,510	17,430	8,050
Sexual contact in the last 3 mo†	No	Yes	Yes	Yes	Yes
Previous infection with dengue virus†	Yes	No	No	No	No
Previous infection with chikungunya virus†	Yes	Yes	No	No	Yes
Fluid Zika-positive by qRT-PCR at baseline	Saliva	Blood, urine	Blood	Urine	Blood, saliva
Vaginal fluid PCR results, C_t_1/C_t_2,‡ at approximate days after symptom onset					
7	NS	NS	NS	0/0	0/0
14	NS	31/0	32/33	NS	0/0
21	NS	35/0	34/36	0/0	37/0
28	33/0	0/0	NS	0/0	NS
60	NS	34/36	NS	36/0	NS
90	NS	35/0	NS	0/0	NS
180	NS	37/0	0/0	NS	NS

*C_t_, cycle threshold; NA, not applicable; NS, no sample; qRT-PCR, quantitative reverse transcription PCR. †By participant report. ‡C_t_1 refers to pan-Zika primers; C_t_2 refers to Asian-specific primers.

## Conclusions

Detection of Zika virus RNA in vaginal secretions 60–180 days after the onset of symptoms is a novel observation; the presence of Zika virus in vaginal secretions for <6 months has crucial implications for couples planning pregnancy and for sexual transmission. In contrast to our results, in a population-based study of patients with symptomatic acute Zika virus infection followed for 6 months, only 1 of 50 women had Zika virus detectable in vaginal secretions, in a single specimen collected 3 dpo (*4*). The longest previously reported persistence of Zika virus RNA in vaginal secretions was 37 dpo (*7*); no other studies have detected Zika virus RNA in the reproductive tract for >14 dpo (*8*). Shedding was intermittent in several patients in our study, so 1 negative specimen does not necessarily signify viral clearance.

Most of our subjects were pregnant at enrollment; pregnant women are known to shed Zika virus in blood for up to 3 times longer than nonpregnant women, possibly because of fetal infection (*9*). Another study reported detection of Zika virus RNA in cervical cytology samples of 32 of 59 pregnant women compared with 18 of 109 nonpregnant women (*10*). Fetal tissue may act as a reservoir for long-term infection, or pregnancy-related immunosuppression might delay viral clearance. One patient in our study (A018) shed Zika virus RNA in the reproductive tract after delivery, suggesting that there may be reservoirs of viral replication in the female reproductive tract. 

Our findings have implications for future pregnancies. Persistent Zika virus shedding in the reproductive tract could be a source of fetal infection. Vaginal shedding of Zika virus could result in ascending infection, as occurs with vaginal cervical herpesvirus 2 infections (*11*), or transmission during delivery, as seen with other viral infections such as human immunodeficiency virus and hepatitis B. Alternatively, transplacental infection may occur during viremia occurring after sexual transmission. Although the role of vaginal replication of Zika virus in fetal infection is not well characterized, mouse models suggest that fetal infection from vaginal sources of Zika virus replication can occur (*12*,*13*).

Our findings also suggest that sexual transmission of Zika virus from women to their sexual partners may be possible for up to 6 months after infection. Infectivity studies are warranted to investigate whether Zika virus shed in vagina secretions is capable of replication and infection. Sexually acquired Zika virus, mainly by male-to-female transmission, has been reported in >18 articles; however, there is evidence of infectious Zika virus particles in vaginal fluids (*14*). We lacked sufficient sample volumes to perform viral culture to distinguish between vaginal shedding of viral RNA and replication-competent virions, a limitation of our study. Only 1 report has documented the presence of culture-confirmed live Zika virus in vaginal secretions from an HIV-infected woman 3 dpo (*14*), and there is 1 reported case of likely female-to-male sexual transmission (*15*). Larger studies are needed to clarify the potential of the female reproductive tract to transmit Zika virus.
